# Supplementary material for: Bayesian differential analysis of cell type proportions: opinion
Source: Front Genet. 2023 Jun 1;14:1205499. doi: 10.3389/fgene.2023.1205499 (PMC10267376; doi:10.3389/fgene.2023.1205499)
Supplement: Supplementary file 1 [file DataSheet1.docx]

**Bayesian Differential Analysis of Cell Type Proportions: Opinion**

Tanya T. Karagiannis^1*^, Stefano Monti^2,3,4^, Paola Sebastiani^1,5^

^1^Institute for Clinical Research and Health Policy Studies, Tufts Medical Center, Boston, MA, USA

^2^ Division of Computational Biomedicine, Boston University Chobanian & Avedisian School of Medicine, Boston, MA, USA

^3^Department of Biostatistics, Boston University School of Public Health, Boston, MA, USA

^4^Bioinformatics Program, Boston University, Boston, MA, USA

^5^Department of Medicine, Tufts University, Boston, MA, USA

**Supplementary Tables**

**Table S1. Table of significant cell type specific credible changes in composition between EL and younger age based on the Bayesian multinomial regression and scCODA.
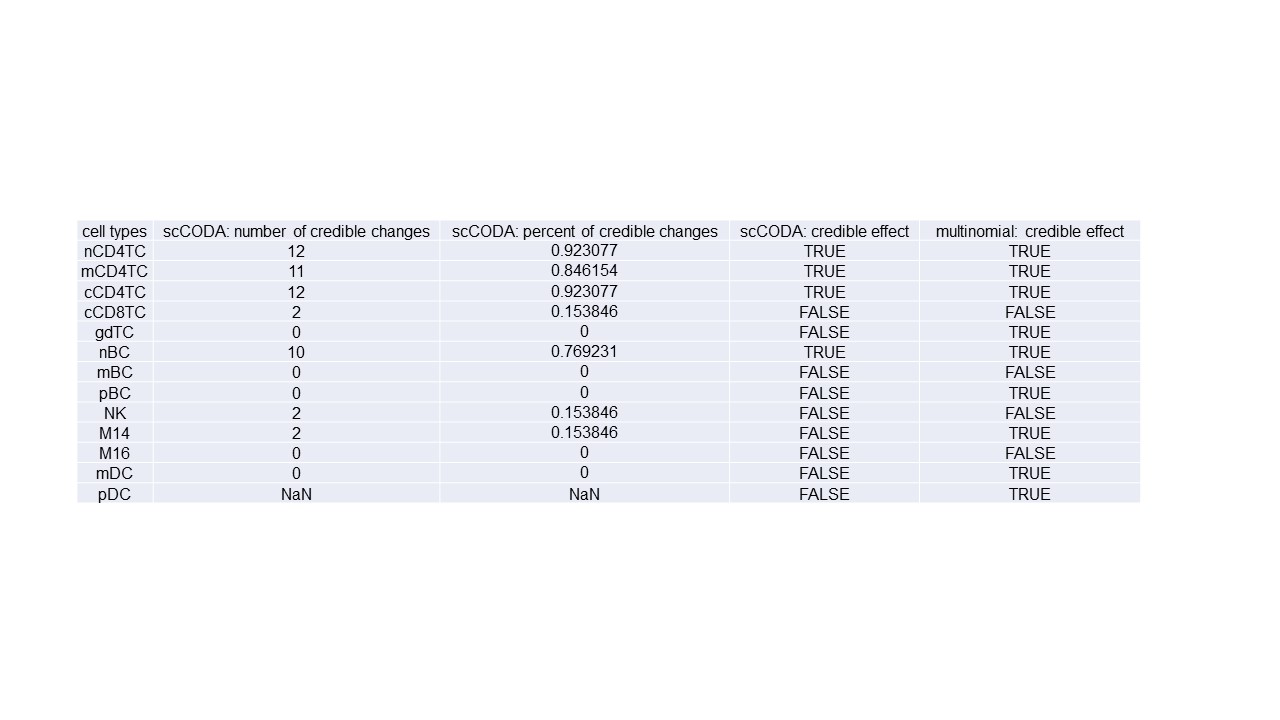
**

**Supplementary Figures**


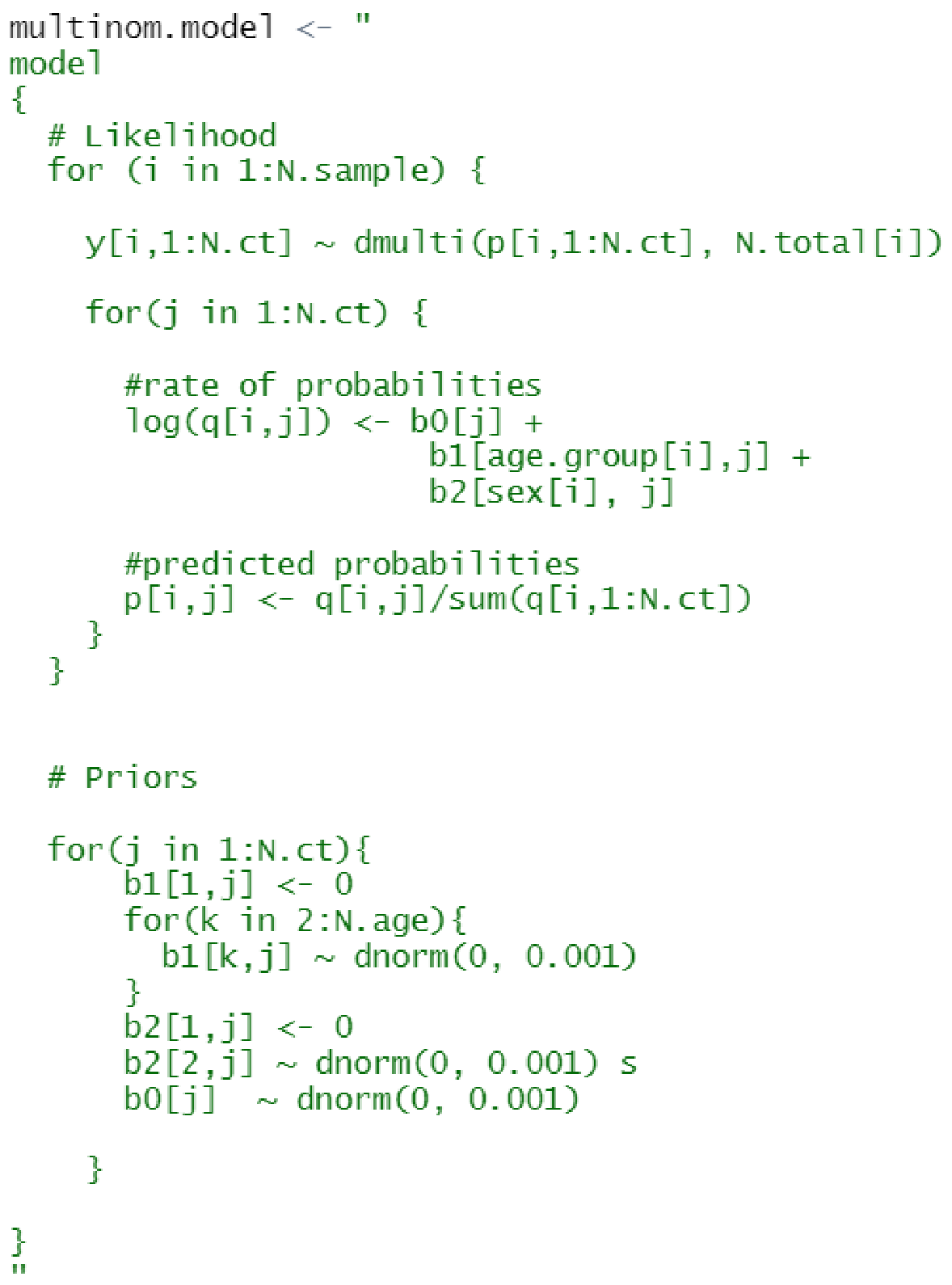


**Figure S1. Bayesian multinomial regression model configuration using rjags package in the R software.** In the model setup, where $Y_{i,1:N.ct}$ denoted as $y\left[ i,1:N.ct \right]$ represents the vector of numbers of cell types $1:N.ct$ in sample $i$ , and is modeled using a multinomial distribution with probabilities $p_{i, 1:N.ct}$ denoted as $p\left[ i, 1:N.ct \right]$ such that $\sum_{j = 1}^{N.ct} Y_{i,j} = {N.total}_{i}$ and $\sum_{j = 1}^{N.ct} p_{i,j} = 1$, for all sample $i.$ The probabilities $p_{i, 1:N.ct}$ can depend on covariates age.group and sexthrough the function $\log\left( q_{i,j} \right)$ denoted as $\log(q[i,j])$. The regression parameters ${\beta0}_{j} , {\beta1}_{{age.group}_{i},j}, {\beta2}_{{sex}_{i},j}$, $j=1:J$ are denoted in the model as $b0\left[ j \right], b1\left[ age.group\left[ i \right], j \right], b2\left[ sex\left[ i \right],j \right]$. To calculate the predicted probabilities of all cell types for each group profile , for each cell type*1:* $j$ with *N.ct*  cell types in total , we can explicitly calculate the estimate of the probability $p_{i,j}$ for each group profile $i$ from the estimates of the parameters ${\beta0}_{j} , {\beta1}_{{age.group}_{i},j}, {\beta2}_{{sex}_{i},,j}$ as shown below:

$$\hat{p}_{i,j} = \frac{exp( \hat{\beta0}_{j} +\hat{\beta1}_{{age.group}_{i}, j} +\hat{\beta2}_{{sex}_{i}, j})}{\sum_{k=1}^{N.ct} exp(\hat{\beta0}_{j} +\hat{\beta1}_{{age.group}_{i}, j} +\hat{\beta2}_{{sex}_{i}, j})}$$

where the notation ^ represents the estimated parameters using rjags.
